# Association between rs11614913 Polymorphism of The *MiR-196-a2* Gene and Colorectal Cancer in The Presence of Departure from Hardy-Weinberg Equilibrium 

**DOI:** 10.22074/cellj.2021.7295

**Published:** 2021-07-17

**Authors:** Ali Reza Soltanian, Bistoon Hosseini, Hossein Mahjub, Fatemeh Bahreini, Nazemalhosseini Mojarad Ehsan, Mohammad Ebrahim Ghaffari

**Affiliations:** 1. Department of Biostatistics, School of Public Health, Hamadan University of Medical Sciences, Hamadan, Iran; 2. Modeling of Noncommunicable Diseases Research Center, Hamadan University of Medical Sciences, Hamadan, Iran; 3. Kermanshah Province Electricity Distribution Company, Kermanshah, Iran; 4. Research Center for Health Center, Hamadan University of Medical Sciences, Hamadan, Iran; 5. Department of Molecular Medicine and Genetics, Faculty of Medicine, Hamadan University of Medical Sciences, Hamadan, Iran; 6. Gastrointestinal (GI) Cancer Department, Gastroenterology and Liver Diseases Research Center, Research Institute for Gastroenterology and Liver Diseases, Shahid Beheshti University of Medical Sciences, Tehran, Iran

**Keywords:** Association, Colorectal Cancer, Equilibrium, Gene Polymorphim

## Abstract

**Objective:**

Colorectal cancer (CRC) is the fourth most common and the second most lethal cancer worldwide. CRC mortality is increasing in Iran. In the current study, we aimed to investigate association between rs11614913 polymorphism of the *miR-196-a2* gene and CRC.

**Materials and Methods:**

In this case-control study, we assessed association of the rs11614913 polymorphism in 194 patients with CRC (case) and 286 healthy individuals (control). The expectation-maximization (EM) algorithm method was used to adjust deviation from Hardy-Weinberg equilibrium (HWE).

**Results:**

There was no significant difference between genotypic frequencies of rs11614913 polymorphism in the control and case groups. Genotypic frequencies differed in the adjusted and unadjusted deviations from the HWE. Analysis of unadjusted and adjusted independent variables showed that age, sex, alcohol consumption, and drug use were statistically significant.

**Conclusion:**

Our findings showed that rs11614913 polymorphism was not associated with CRC risk. Deviation from HWE affected the results. It is recommended to perform further studies to establish HWE. Ignoring the equilibrium can cause in consistencies in the results of studies.

## Introduction

Non-communicable diseases (NCDs) are now responsible for most of deaths worldwide (1). Cancer is predicted to be the leading cause of death and the most important obstacle to increaselife expectancy in the 21st century (2). Increasing the burden of cancer and other NCDs are treats of human development (3). Colorectal cancer (CRC) is the fourth most common cancer type worldwide (2). It is an important public health problem in different populations (4). The World Health Organization (WHO) predicted that by the year 2030 frequency of new cases of CRC and its related deaths will increase by 77 and 80%, respectively. It was shown that frequency of CRC related deaths was increased within the past 10 years in Asia (5). Although it is less prevalent in the Middle East, studies indicateda growing trend. In Iran, as a region in the Middle East, this trend is increasing (6). Although the exact causes of CRC are yet unknown, it was shown that CRC is influenced by environmental and genetic factors (7).

In recent years, role of predisposing genetic factors in mediating tendency of CRC became more and more apparent (8). Recently, a growing number of studies focused on the association of microRNA (miRNA) polymorphisms with cancer susceptibility, suggesting that accumulation of genetic variants may be involved in cancer progression (9). *miR-196a2* rs11614913, as a definitional miRNA polymorphism, is crucially associated with cancer risk (10). Previous studies showed that *miR-196a2* rs11614913 polymorphism is associated with susceptibility to cancer, especially in lung cancer and hepatocellular carcinoma as well as head and neck cancer. The *miR-196a2* rs11614913 polymorphism may act as a risk factor for cancer patients (9).

Identification of genetic polymorphisms associated with cancer has many diagnostic implications. For example, early detection of high-risk individuals may be possible, which in turn can take different measures to reduce the risk of cancer development or progression (8). There are limited studies on the association of the *miR-196-a2* polymorphism with CRC andthe results are inconsistent (11-13). In Iran, only one study with a small sample size was conductedin this regard without Hardy-Weinberg equilibrium (HWE) calculation (14). According to importance of genetic studies in the early detection of CRC and the importance of considering internal validity, the present study was performed to investigate association of rs11614913 polymorphism of the *miR-196-a2* gene and CRC in the presence of departure from HWE.

## Materials and Methods

### Subjects

This hospital-based case-control study was conducted in Taleghani Hospital (Tehran, Iran) from 2014 to 2019. A total of 194 patients with histologically confirmed CRC and without family history of related cancers were enrolled in this study. Two hundred and eighty-six individuals with no colonoscopy signs of CRC were randomly selected from the same residential areas as control group. The study was approved by the Hamadan University of Medical Sciences Ethics Committee (IR.UMSHA.REC.1396.640) and all participants provided a written informed consent. The participants were interviewed and data on gender, age, smoking, alcohol consumption and addiction (opium) were obtained using a structured questionnaire. 

### DNA extraction

Genomic DNA was extracted using a standard salt extraction protocol (15, 16). Polymerase chain reaction-restriction fragment length polymorphism (PCR-RFLP) method was used to determine the genotype distributions of *miR-196-2* polymorphism (rs11614913). The specific primers were designed using Primerblast (http://www. ncbi.nlm.nih.gov/tools/primer-blast/) and the software Gene Runner. The primers sequences were:

F: 5ˊ-GTCTACTCTCTAGTCCTTAGG-3ˊ

R: 5ˊ-TTGAGAGGACGGCATAAAGC-3ˊ

The primers amplified a 383 base pair (bp) fragment. The PCR was done in a final volume of 25 μl with 35 temperature cycles consisting of 45 seconds at 94˚C (denaturation), 40 seconds at 55.2˚C (annealing) and 45 seconds at 72˚C (extension). A final extension step at 72˚C was performed for 5 minutes. The PCR products were exposed to restriction enzymes HpyCH4III for 8 hours at 37˚C. The enzyme digested products were visualized on 3% agarose gels and stained with DNA green viewer (for visualization under a UV light). Extracted DNA samples were amplified for the segment that comprised rs11614913 polymorphism. Agarose gel (1%) electrophoresis was done to confirm the PCR product size. The expected product size was 383 bp, which was confirmed by 1% agarose gel electrophoresis.The results of 2% agarose gel electrophoresis on PCR products were digested by restricted endonuclease HpyCH4III.

At this single nucleotide polymorphism (SNP), the nucleotide C is converted to T.

5´... A C N▼G T ... 3´

3´... T G▲N C A ... 5´

The HpyCH4III enzyme cut site is shown in the above. This enzyme cuts the rare T genotype. In other words, in the normal case, it contains nucleotide C that the enzyme does not recognize and does not cut. In this case, if we have C in one allele and T in the other allele, the condition is heterozygote. To confirm the genotyping results, we selected 10% polymerase chain reaction products for DNA sequencing and the results were 100 % concordant.

### Statistical analyses

### Method of adjusting the deviation from Hardy-Weinberg equilibrium

Chi-square test was used to evaluate HWE in the control group. Regarding the absence of HWE (P=0.046), the expectation-maximization (EM) algorithm method was used to adjust deviation from HWE (17). For this purpose, the level of deviation was assessed using EM algorithm in the estimation of allele frequency. Then, the genotype frequencies were estimated. For the estimation of allele frequency, disequilibrium coefficient (D) was set for the population deviation from equilibrium. This value is due to the difference between the true value of genotype and the expected value under equilibrium: with the selection of PA(0) for the frequency of allele A in the step E of the algorithm, the expected number of genotypes was obtained from equation 1.

$$n_{\mathrm{AA(m)}}=\frac{P_{\mathrm{A(m-1)}}^{2}+D}{P_{\mathrm{A(m-1)}}^{2}+2P_{\mathrm{A(m-1)}}(1-P\mathrm{A(m-1)})D}n_{A}$$

In the step M, the new value of the allele frequency was obtained using equation 2.

pA(m)^=2nAA(m)+nAa(m)2n

If |PA(m)^-PA(m)^PA(m-1)^|≤0.01 or m≥100 (m is the repetition of the algorithm) or PA(m)^=0, the algorithm was stopped and the value of PA(m)^ was considered as the = 0 final value for the frequency of allele A (17).

### Tests and software

Simple logistic regression analysis was used to determine the effect of each independent variable on CRC and multiple regression analysis was performed for adjusting any confounding variables. Chi-square test and logistic regression analyses were used to investigate the association between rs11614913 polymorphismand CRC. Version 16.0 of the SPSS software (SPSS, Inc., USA) and version 3.4.3 of the R software (to adjust the deviations from equilibrium) were used to perform statistical analysis. A P<0.05 was considered statistically significant.

## Results

Demographic characteristics of the studied subjects (286 controls and 194 CRC cases) are shown in Table 1.

Results of the simple and multiple logistic regression analyses are shown in Table 2. The oddsof CRC in men was 1.82 times more than women. The odds of CRC in subjects with drug (opium) use, alcohol consumption and smoking were also respectively 12.74, 7 and 2.43 times more than those who did not use any of them. Analysis of different age groups revealed that the odds of CRC in subjects with age over 50 years was 12.52 times higher than those with age under 50 years. In adjusted analysis, age and sex groups were significantly associated with CRC, so that the odds of CRC in men was 2.38 times higher than women and in subjects with age over 50 years, it was 13.84 times higher than those with age under 50 years. In addition, the odds of CRC in subjects with alcohol and drug consumption were respectively 3.44 and 2.88 fold higher than those who did not, while the differences were notstatistically significant. According to the classification of effect size for the odds ratio, which is 1.5 small, 2 medium and 3 large (18), alcohol and drug use have a significant impact on CRC.

Genotype distribution of the rs11614913 polymorphism for *miR196a2* in patients with CRC was as follows: the homozygous CC genotype was detected in 74 subjects (38.1%), the homozygous TT genotype in 29 subjects (15%) and the heterozygous CT genotype in 91 subjects (46.9%). The genotype distribution of the rs11614913 polymorphism in control group was as follows: the homozygous CC genotype was detected in 108 subjects (37.8%), the homozygous TT genotype in 56 subjects (19.6%) and the heterozygous CT genotype in 122 subjects (42.6%). Regarding the absence of HWE in the control group (P=0.046), the EM algorithm method was used to adjust deviation from HWE. The adjusted genotype distribution of the rs11614913 polymorphism in the control group is shown in Table 3.

T and C allele frequencies were 40.91 and 59.09%, respectively, in the patient group, while they were respectively 38.4 and 61.6% in the control group. Genotype distribution of the rs11614913 polymorphism of the *miR196a2* gene in patients and control groups are shown in Table 3. After adjusting deviation from the equilibrium, frequency of CC, CT and TT genotypes observed in prototype 108 (37.8%), 122 (42.6%) and 56 (19.6%) were changed to 100 (35%), 138 (48.2%) and 48 (16.8%), respectively. The risk of CRC in subjects with CC genotype was higher than those with CT and TT genotype, but the differences were not significant. 

**Table 1:** Demographic characteristics of the studied subjects


Variable		Control (n=286)	Case (n=194)

Gender			
	Male	121 (52.2)	111 (47.8)
	Female	165 (66.5)	83 (33.5)
Age (Y)			
	<50	172 (89.1)	21 (10.9)
	>=50	104 (39.5)	159 (60.5)
Smoking			
	No	263 (62.2)	160 (37.8)
	Yes	23 (40.4)	34 (59.6)
Alcohol consumption			
	No	282 (62.3)	171 (37.7)
	Yes	4 (19)	17 (81)
Opiumuse			
	No	285 (61.4)	176 (38.6)
	Yes	1 (11.1)	8 (88.9)


Data are presented as n (%).

**Table 2:** Association of independent variables in unadjusted and adjusted form with dependent variable (CRC=yes, CRC=no)


Variable		Unadjusted^a^		Adjusted^b^	
		OR (CI 95%)	Significance	OR (CI 95%)	Significance

Gender					
	Male	1		1	
	Female	0.55 (0.38 – 0.79)	0.001	0.42 (0.26 – 0.67)	<0.001
Smoking					
	No	1		1	
	Yes	2.43 (1.38 – 4.27)	0.002	0.92 (0.40 – 2.13)	0.842
Alcohol consumption					
	No	1		1	
	Yes	7 (2.32 – 21.17)	0.001	3.44 (0.75 – 15.72)	0.112
Opiumuse					
	No	1		1	
	Yes	12.74 (1.58 – 102.70)	0.017	2.88 (0.24 – 33.87)	0.401
Age (Y)					
	<50	1		1	
	>=50	12.52 (7.47 – 20.98)	<0.001	13.84 (8.05 – 23.78)	<0.001


^a ^; Simple logistic regression, ^b^ ; Multiple logistic regression, OR; Odd ratio, and CI; Confidence interval.

**Table 3:** The genotype and allele distribution of the rs11614913 polymorphism in the case and control groups by adjusting deviation from the HWE to the control group


Genotype	Casen (%)	Controln (%)	OR, 95% CI (Lower-Upper)	P value (χ2, df)	Adjusted OR, 95% CI (Lower-Upper)	Adjusted P value (χ2, df)	Minimum detectable OR

RS116							
CC	74 (38.1)	100 (35)	Reference group	-	Reference group	-	-
CT	91 (46.9)	138 (48.2)	1.09 (0.73 – 1.63)	0.358 (0.85, 1)	0.89 (0.60 - 1.33)	0.772 (0.08 , 1)	0.57
TT	29 (15)	48 (16.8)	0.76 (0.44 – 1.29)	0.192 (1.70, 1)	0.82 (0.47 – 1.42)	0.591 (0.29 , 1)	0.46
CT+TT	120 (61.9)	186 (65)	0.98 (0.68 – 1.43)	0.980 (0.001, 1)	0.87 (0.60 – 1.27)	0.477 (0.51 , 1)	0.58
Alleles							
C	239 (61.6)	338 (59.09)	Reference group	-	Reference group	-	-
T	149 (38.4)	234 (40.91)	0.90 (0.69 – 1.17)	0.436 (0.61, 1)	0.90 (0.69 – 1.17)	0.436 (0.61, 1)	0.69


HWE; Hardy-Weinberg equilibrium, OR; Odd ratio, and CI; Confidence interval.

## Discussion

In the present study, we found that the genetic variant rs11614913 polymorphism of the *miR-196-a2* was not significantly associated with CRC. This lack of association was obtained either using the raw control genotype data or the artificially adjusted distribution followed to fit HWE. It is important to consider the degree of deviation from the equilibrium. Based on STrengthening the REporting of Genetic Association studies (STREGA) guideline, if HWE is not maintained, the results will be wrong. Despite the significance of this equilibrium, there are still many other genetic association studies which do not notice it (19, 20).

Accuracy of the results reported in a variety of studies (e.g. CONSORT, STROBE) depends on the internal validity of the study. In 2009, Little et al. (21) provided an extension of the STrengthening the Reporting of OBservational Studies in Epidemiology (STROBE) guidelines as titled STREGA to assess internal validity of genetic association studies. One of the STREGA items is about HWE. Based on STREGA guideline, if HWE is not maintained, the results will be wrong. The STREGA guideline did not explain a solution when there is deviation from the equilibrium. For this reason, in this paper, we used a new applied method to adjust the deviation from the equilibrium. These results have represented that if the HWE is not balanced in the control group, it can have an impact on the results of the study.

The occurrence of several mutations at tumor suppressor genes or proto-oncogenes is the critical genetic cause of CRC development (22). micro-RNAs can act as an oncogene or oncomiR through inhibition of cancer-related genes. Therefore, micro-RNAs can be studied as possible biomarkers for the diagnosis of cancer. To date, several studies showed association of *miR196a2* rs11614913 polymorphism with various malignancies (9). Hu et al. (23) reported in 2008 that risk of non-small cell lung cancer (NSCLC) is higherin individuals with homozygous CC genotype of rs11614913 polymorphism of the *miR196a2* and the prognosis of NSCLC is worse in these patients. Additionally, it was reported that risk of breast cancer is lowerin individuals with the homozygous TT genotype (24). However, results of the previous study, regarding the effect of *miR196a2* rs11614913 polymorphism on CRC are challenging (13). In the present study, allele and genotype frequencies of rs11614913 polymorphism of the *miR196a2* gene were assessed in Iranian patients with CRC to find the possible association between the rs11614913 polymorphism as a genetic factor and CRC. Our results showed that risk of CRC in subjects with CC genotype was higher than subjects with CT or TT genotypes. In addition, risk of CRC in subjects with the C allele was more than subjects with the T allele, but the difference was not significant. Our results are similar to the two other studies that studied CRC. They also did not find any significant association between *miR-196a2* polymorphism and the risk of CRC was found. Hezova et al. (12) investigated 197 patients with non-hereditary CRC and 212 control subject in Europe. They did not find any correlation between the rs11614913 polymorphism of the *miR196a2* gene and the risk of CRC. Their finding was consistent with the data obtained from Chen et al. (11). Previous study obtained in Iran showed a significant association without equilibrium calculation (14).

On the other hand, Zhan et al. (13) reported that the C allele of rs11614913 polymorphism is a risk factor for CRC. However, they did not find any association between rs11614913 polymorphism and factors such as tumor size, cancer stage and metastasis. Notably, another study reported that risk of gastric cancer in Chinese individuals with the CC genotype of *miR196a2* is higherthan those with CT and TT genotype. Therefore, the C allele of rs11614913 polymorphism has a considerable effect on gastrointestinal cancer in China (25).

Our results showed that risk of CRC, with both unadjusted and adjusted form, was higherin subjects with age over 50 years. In 2019, Wong et al. (26) showed that age is a risk factor for CRC. In line with the present study, it was shown that risk of CRC was increased dramatically after age 50 years; 90% of all CRCs were diagnosed after 50 years old. Our results also showed that the risk of CRC in men was 2.38 times higher than women. Previous studies performed by Wong et al. (26) and Kolligs et al. (27) found similar results among the advanced cancer patients. In addition, in the unadjusted analysis, results of the present study showed that risk of CRC was high in subjects who were using alcohol, drug and smoke. In the adjusted analysis, alcohol and drug use had a significant impact on CRC. It was reported that CRC (∼30-50%) was affected by lifestyles, such as a high red and processed meat consumption, obesity, diabetes and alcohol overuse (26). It was postulated that smoking is responsible for 12% mortality of CRC. Carcinogenic substances in tobacco smoke increase risk of colorectal cancer. By comparing with non-drinkers, it was showed that higher alcohol consumption was significantly associated with elevated CRC risk (28, 29). 

## Conclusion

Our results showedthat with and without using EM method, no significant association did exist between rs11614913 polymorphism and CRC risk. Deviation from HWE affected the results. It is suggested that future studies of this polymorphism should investigate HWE. Ignoring the equilibrium can cause inconsistencies in the results of studies.
